# York County Hospital: A Sister's View

**Published:** 1913-04-12

**Authors:** E. M. Hill

**Affiliations:** Canterbury


### THE EDITOR'S LETTER-BOX.

### YORK COUNTY HOSPITAL: A Sister's View.

*To the Editor of* THE HOSPITAL.

SIR,—Having been a sister of the York County Hospital for over two years and resigned quite recently, I beg to emphatically protest against Doctors Macqueen's and Shepherd's accusations of ill-treatment of probationers by the matron and sisters. They are treated with the greatest kindness and consideration. Complaints of illness were never ignored, but at once reported. In my case I did not wait for complaints, but if a nurse showed any symptom of illness at once took the necessary measures, and frequently she was invited to rest in my own private room till further arrangements were made. I am quite sure my fellow-sisters did the same. I had several sick nurses in my private ward, and they were in every respect nursed with the utmost attention.

Are Doctors Macqueen and Shepherd not aware that a keen nurse is not anxious to go off duty for every little ailment, lest she miss her opportunities of training, her lectures, and examinations?

Are they not aware also that it is not etiquette for young inexperienced resident officers to attend the nursing staff in illness? They were always attended by the honorary staff at York, as is usual elsewhere.

I have had letters from two probationers still in training, telling me how happy they have been at York, and expressing satisfaction with their treatment and the kindness of the matron towards them.

The food at York is quite up to the average of general hospitals. As far as the doctors' meals are concerned, in several instances paying patients in my ward have had dishes from the residents' dinner-table for their evening meal, and have been more than pleased with the game, nicely prepared fish, creams, etc., which came up. These ladies were suffering from nervous diseases, a class of patients always fastidious about their food.

Personally, I worked smoothly with both men, though their work was not done to admiration. As to the graver charges—none of which affect me—both doctors well know that they have been thoroughly dealt with already, as the inquiry which is about to take place will show.

If in their short four and seven months respectively of office Dr. Macqueen and Dr. Shepherd had applied themselves whole-heartedly to their work, instead of resorting to such childish tricks as smashing china, tearing off the wall-paper, and trying to stir up discontent among the probationers, they might have some little right to talk of "raising the tone" of the hospital.

Since Sir Henry Burdett's report was published from five to eight months before either men were in residence, it seems strange that they should venture to come to so terrible a place!—Yours faithfully,

Canterbury, April 4. E. M. HILL.

### Dr. Macqueen's Reply.

*To the Editor of* THE HOSPITAL.

